# Xuan Bi Tong Yu Fang Promotes Angiogenesis via VEGF-Notch1/Dll4 Pathway in Myocardial Ischemic Rats

**DOI:** 10.1155/2020/5041629

**Published:** 2020-02-05

**Authors:** Shuangdi Li, Jingrong Dong, Guang Ta, Yinghui Liu, Junfeng Cui, Xiaohui Li, Jing Song, Aidong Liu, Guangyu Cheng

**Affiliations:** ^1^Center of Heart Disease, Affiliated Hospital of Changchun University of Traditional Chinese Medicine, Changchun, China; ^2^Department of Endoscopy, Affiliated Hospital of Changchun University of Traditional Chinese Medicine, Changchun, China; ^3^Department of Intensive Care Unit, Affiliated Hospital of Changchun University of Traditional Chinese Medicine, Changchun, China; ^4^Changchun University of Traditional Chinese Medicine, Changchun, China; ^5^President's Office, Affiliated Hospital of Changchun University of Traditional Chinese Medicine, Changchun, China; ^6^The Third Affiliated Hospital of Changchun University of Traditional Chinese Medicine, Changchun, China; ^7^Experimental Research Center, Affiliated Hospital of Changchun University of Traditional Chinese Medicine, Changchun, China

## Abstract

**Objective:**

To investigate the effect of Xuan Bi Tong Yu Fang (XBTYF) on angiogenesis via the vascular endothelial growth factor- (VEGF-) Notch1/delta-like 4 (Dll4) pathway. *Materials and Methods*. Sixty Sprague-Dawley rats were randomly divided into six groups: control, sham-operated, myocardial ischemia model, and XBTYF treatment at 3.2, 1.6, and 0.8 g/kg. Electrocardiography was performed to evaluate the successful establishment of the model. Hematoxylin-eosin staining and transmission electron microscopy were carried out to observe the morphology and mitochondrial structure in myocardial cells, respectively. TUNEL staining was performed to assess the degree of cell apoptosis. The expression of VEGF-A, Notch1, Dll4, Bcl2, Bax, caspase 3, caspase 9, and cytochrome-c (Cyt-c) was observed by western blot.

**Results:**

XBTYF inhibited changes to the morphology and mitochondrial structure in cardiomyocyte and reduced cell apoptosis. Compared with the model group, XBTYF at all doses (3.2, 1.6, and 0.8 g/kg) reduced the expression of Notch1, Dll4, Bax, caspase 3, caspase 9, and Cyt-c, whereas expression of VEGF-A and Bcl2 was increased.

**Conclusion:**

XBTYF attenuated mitochondrial damage and cell apoptosis while promoting the angiogenesis of cardiomyocyte. The associated mechanism may be related to the VEGF-Notch1/Dll4 pathway.

## 1. Introduction

Coronary heart disease (CHD) is characterized by myocardial ischemia, hypoxia, and necrosis. The “China Cardiovascular Disease Report 2017” [1] pointed out that cardiovascular diseases accounted for more than 40% of disease-related deaths among residents in China, which was much higher than the incidence of tumors and other diseases. In addition, the morbidity and mortality of cardiovascular diseases are still on the rise. CHD has one of the highest mortality rates worldwide and is a high-risk condition among cardiovascular diseases. According to a report of the World Health Organization in 2011, China's death toll from coronary heart disease ranks second in the world. Coronary artery bypass grafting is the main treatment for coronary artery disease, but as it is an invasive technique (more complications) and incurs a high cost, its clinical application is affected. The main pathological feature of coronary heart disease is coronary artery stenosis, which leads to myocardial ischemia. Therefore, therapeutic angiogenesis has gradually become highlighted in the recent 10 years. Therapeutic angiogenesis promotes the release of proangiogenic factors from the ischemic myocardium through certain interventions, forming new small blood vessels and establishing effective collateral circulation near the ischemic myocardium and thereby restoring blood supply to the ischemic myocardium and improving patient symptoms. Nowadays, many novel methods have been invented to treat CHD. Krueger et al. [2] found that 3D GF scaffolds significantly promote the growth and differentiation of C2C12 myoblasts. Shin et al. [3] showed that 3D GO-coated PU foams strongly evoked spontaneous myogenic differentiation of C2C12 skeletal myoblasts without any myogenic factors. Regretfully, many studies have reported [4–7] that 3D GFs containing a significant fraction of cross-linked, single-layer graphene sheets with extremely sharp edges can physically damage the wall membrane of cells. As its potential toxicity, probable long-term side effects, and biocompatibility [8], it is still not an ideal CHD treatment strategy.

The vascular endothelial growth factor (VEGF) gene family includes VEGF-A, VEGF-B, VEGF-C, VEGF-D, VEGF-E, and PIGF. Our commonly known VEGF refers to VEGF-A, and VEGF-A has four subtypes: VEGF_121_, VEGF_165_, VEGF_189_, and VEGF_206_. VEGF plays a critical role in the regulation of angiogenesis [9] and promotes the proliferation, differentiation, and migration of vascular endothelial cells, increases vascular permeability, and inhibits apoptosis. Vascular endothelial cells can express Notch1, Notch4, delta-like 4 (Dll4), etc. Dll4 is an important vascular growth regulator who binds with Notch1 to regulate angiogenesis. Chen et al. [10] found that VEGF enhances ETS1-BRD4 interaction to broadly promote RNAPII pause release and drive angiogenesis. Niderla-Bielińska et al. [11] reported that sulodexide inhibits angiogenesis via decreasing Dll4 and Notch1 expression.

Thousands of years of clinical practice have accumulated a considerable number of single herbs or herbal formulas that exhibit safety and efficacy in treating CHD. Xuan Bi Tong Yu Fang (XBTYF), a famous traditional Chinese medical prescription, is composed of corydalis yanhusuo, notoginseng, Ligusticum Chuanxiong, Tulip, Ginseng, and Borneolum Syntheticum using methods of traditional Chinese medicine preparation. Our previous experiments demonstrated that XBTYF protected the heart of myocardial ischemic rats by promoting angiogenesis [12, 13], but the underlying mechanism of XBTYF involved within is still unclear. In this study, we established the myocardial injury model to explore the effect of XBTYF on the VEGF-Notch1/Dll4 signaling pathway and angiogenesis.

## 2. Materials and Methods

### 2.1. XBTYF Preparation and Animals

XBTYF was prepared from Corydalis yanhusuo, notoginseng, Ligusticum Chuanxiong, Tulip, Ginseng, and Borneolum Syntheticum using methods of traditional Chinese medicine procedures. Sixty healthy Sprague-Dawley rats (half male and half female, 200–220 g, 8 weeks) were purchased from the Animal Experimental Center of Changchun University of Chinese Medicine (SCXK 2015-0001). The rats were randomly divided into 6 groups (*n* = 10 per group): control, sham-operated, myocardial ischemia model, XBTYF at the high (3.2 g/kg), medium (1.6 g/kg), and low (0.8 g/kg) doses according to the previous research [12]. Rats were cultured in a specific pathogen-free environment at a temperature of 22°C–26°C with relative humidity of 50–60% in a 12/12-h light/dark cycle and were fed standard feed and pure water. All animals used in this study were approved by the Animal Care and Use Committee, Wuhan hualian biotechnology co. LTD. (HZAURA-2018-010).

### 2.2. Construction of the Myocardial Ischemia Model

The myocardial ischemia model was constructed according to the methods reported by Liu et al. [14]. The rats were subjected to adaptive feeding for 7 days and anesthetized by intraperitoneal injection of 50 mg/kg pentobarbital (P3761, Sigma). The rats were then fixed with rubber bands in a supine position on a plate and connected to an eight-lead data recorder to obtain normal electrocardiograms before thoracotomy. The rats were tracheotomized, intubated, connected to a small-animal ventilator, and ventilated continuously for 10 × 60 ml/min. Next, the rats were depilated and disinfected with iodine alcohol. The thoracic cavity was opened, the pericardium was cut, the heart was squeezed out, the left coronary artery was ligated with line 4, and the secretions and suture wounds were cleared. The sham-operated rats were only subjected to the operative line without ligation. Electrocardiography was performed to evaluate the successful establishment of the model. After the operation, 40,000 units of penicillin were injected intramuscularly per day for three days to prevent infection. Intragastric administration of XBTYF was performed once a day for 4 weeks the day after the model was successfully constructed, at XBTYF doses of 3.2 g/kg (high), 1.6 g/kg (medium), and 0.8 g/kg (low). The control and sham-operated rats were treated with 0.9% normal saline.

### 2.3. Hematoxylin-Eosin (HE) Staining

The myocardial tissue microsections were hydrated and placed in hematoxylin for 5 min and 0.5% eosin staining solution for 3 min. The sections were mounted with neutral glue and observed using a microscope (MD1000, Leica, Solms, Germany).

### 2.4. Transmission Electron Microscopy (TEM)

Samples were prefixed with 2.5% glutaraldehyde (10–20 times the tissue volume) at 4°C for 30 min, fixed with 1% osmic acid for 1 h, dehydrated, soaked in a 1 : 1 mixture of acetone:epoxy at 40°C for 6 h, fixed with pure epoxy resin at 40°C for 4 h, and embedded. The samples were then sliced and subjected to double staining and lead citrate staining for 15 min. After rinsing with double-distilled water, the ultrastructure of the mitochondria was observed using TEM (HT7700, Hitachi, Tokyo, Japan).

### 2.5. Terminal Deoxynucleotidyl Transferase dUTP Nick End Labeling (TUNEL)

Paraffin-embedded tissue specimens were sliced and underwent conventional dewaxing and hydration. The sections were placed into 3% hydrogen peroxide methanol for 15 min, and each sample was incubated with 100 *μ*l of protease K (20 *μ*g/ml) for 20 min. After two washes with phosphate-buffered saline for 5 min each, the samples were incubated with TUNEL reaction solution at 37°C for 1 h. The converter-POD was then added to the samples in the cassette. After three rinses, the samples were stained with diaminobenzidine (ZLI-9018, ZSGB-BIO Co., Ltd., China) and hematoxylin. The sections were sealed, dried, and observed using an optical microscope (MD1000, Leica, Solms, Germany). The positive granules were assessed in Image Pro-Plus 6.0.

### 2.6. Western Blot

Myocardial tissues stored at −80°C were removed and lysed using radioimmunoprecipitation assay buffer lysate in an Eppendorf tube at 50 *μ*g of tissue per ml. The lysates were centrifuged at 3,000 rpm for 5 min at 4°C, subjected to ultrasound three times for 5 min each, and centrifuged at 15,000 rpm for 5 min. The protein concentration of the extracted supernatant was measured using a commercial bicinchoninic acid kit (Beyotime, China) according to the manufacturer's instructions. Total protein was separated by 12% sodium dodecyl sulfate-polyacrylamide gel electrophoresis and transferred to polyvinylidene fluoride membranes. The membranes were blocked for 120 min in blocking buffer, washed five times with Tris-buffered saline (TBS)/0.1% Tween 20, and probed with primary antibodies against VEGF-A (1 : 1000, PAB30096, Bioswamp), Notch1 (1 : 1000, PAB35376, Bioswamp), Dll4 (1 : 1000, ab7280, Abcam), Bcl2 (1 : 1000, PAB30041, Bioswamp), Bax (1 : 1000, PAB30040, Bioswamp), caspase 3 (1 : 1000, 19677-1-AP, Sanying), caspase 9 (1 : 1000, 10380-1-AP, Sanying), Cyt-c (1 : 1000, 12245-1-AP, Sanying), and GAPDH (1 : 2000, PAB36264, Bioswamp) overnight at 4°C. The membranes were washed three times with TBS/0.1% Tween 20 and incubated with secondary antibody (goat anti-rabbit IgG, 1 : 20000, Bioswamp) for 60 min. Protein bands were visualized by enhanced chemiluminescence color detection (Tanon-5200, TANON) and analyzed using AlphaEase FC gel image analysis software.

### 2.7. Statistical Analysis

Data are expressed as the mean ± standard deviation. To analyze the differences between groups, data comparison was performed by *t*-tests and one-way analysis of variance using SPSS 22 statistical software. *P* < 0.05 was considered statistically significant.

## 3. Results

### 3.1. Myocardial Injury Model Validation

**Figure 1:**
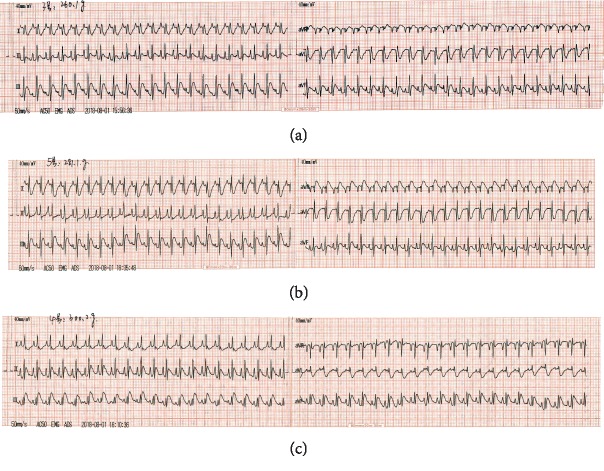
Electrocardiograms of (a) control, (b) sham-operated and (c) model rats. Compared with the control group and sham-operated group, ST segment in the model group increased significantly in II, III and aVF.

We used electrocardiography to validate the successful establishment of the myocardial injury model. From Figure 1, we observed that rats in the model group showed significant increases in ST segment in II, III, and aVF compared with those of the control and sham-operated rats, indicating that the myocardial injury model was constructed successfully.

### 3.2. XBTYF Inhibited Morphological Changes in Cardiomyocyte

**Figure 2:**
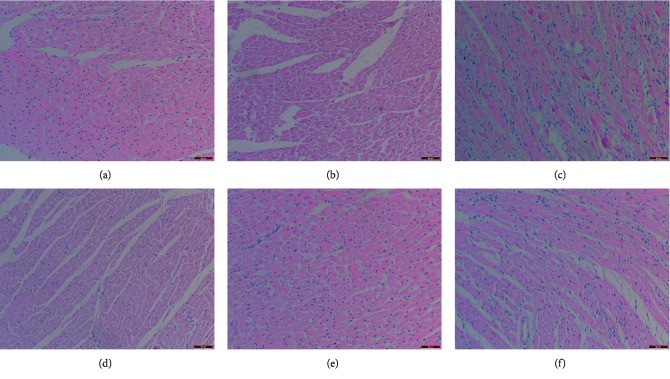
Myocardial morphology was observed by HE staining (scale bar = 50 *μ*m). Myocardial cells in the control and sham-operated rats were neatly arranged and uniform, without red ischemic changes. Myocardial cells in the model rats were arranged with ambiguous disorder, accompanied by inflammatory cell infiltration, large fiber deposition area, and vascular fibrocyte proliferation. With the increase of XBTYF concentration, inflammatory cell infiltration and vascular proliferation gradually decreased, and the morphology of myocardial cells in high-dose group was similar to that in the control group. (a) Control. (b) Sham-operated. (c) Model rats. (d) XBTYF 3.2 g/kg. (e) XBTYF 1.6 g/kg. (f) XBTYF 0.8 g/kg.

From Figure 2, we observed that the myocardial cells in the control and sham-operated rats were neatly arranged and uniform, without red ischemic changes. Myocardial cells in the model rats were arranged with ambiguous disorder, accompanied by inflammatory cell infiltration, large fiber deposition area, and vascular fibrocyte proliferation. When XBTYF was administered at the high doses (3.2 g/kg), only a small number of inflammatory cells were infiltrated and blood vessels proliferated slightly. The effect of low-dose XBTYF (0.8 g/kg) was the poorest because of the presence of inflammatory cell infiltration, large fiber deposition area, and vascular fibrocyte hyperplasia.

### 3.3. XBTYF Inhibited Mitochondrial Damage in Cardiomyocyte

**Figure 3:**
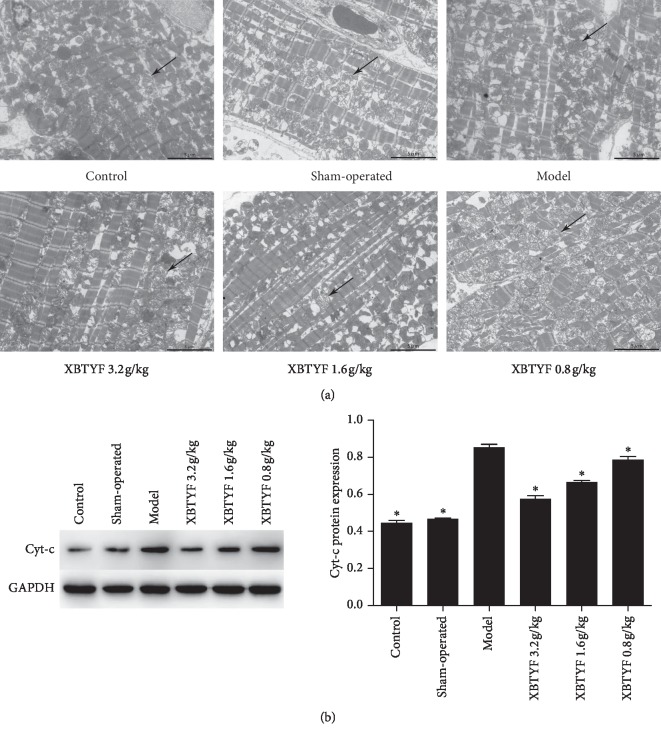
Characterization of mitochondria in cardiomyocytes. (a) Mitochondrial morphology was observed by TEM (scale bar = 5 *μ*m). Arrows indicate the mitochondria of cardiomyocytes. (b) Cyt-c protein expression was detected by western blot. Compared with the model group, the expression of Cyt-c in XBTYF-dose group (3.2, 1.6, and 0.8 g/kg) were significantly decreased, with high-dose group showing the greatest effect. Values represent the average of three replicates. ^*∗*^*P* < 0.05 vs. model.

The mitochondrial structures in myocardial cells (Figure 3(a)) in the control and sham-operated rats were intact with intimal ridges and orderly arrangement. In the model group, the mitochondrial membranes were ruptured and swollen, and the inner cristae were ruptured. The mitochondrial membrane structure in the high-dose XBTYF group was relatively intact, and the inner cristae had a small amount of fracture and dissolution. In the medium- and low-dose XBTYF groups, a small amount of rupture and swelling of the mitochondrial membrane and inner crista rupture were observed. We also evaluated the expression of Cyt-c (Figure 3(b)), the data were normally distributed (*P* > 0.05), and results showed that it was significantly downregulated by XBTYF at all doses (3.2, 1.6, and 0.8 g/kg) (*P* < 0.05), with high dose showing the greatest effect.

### 3.4. XBTYF Reduced Cardiomyocyte Apoptosis

**Figure 4:**
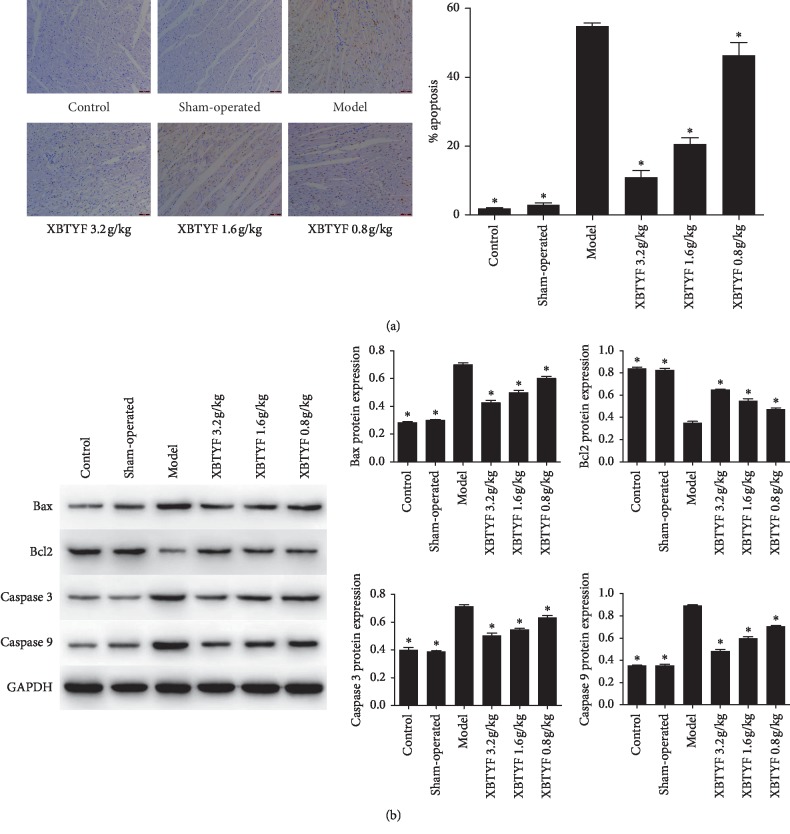
Evaluation of cardiomyocyte apoptosis after XBTYF treatment. (a) Cardiomyocyte apoptosis was detected by TUNEL assay (scale bar = 50 *μ*m). Compared with the model group, cell apoptotic rate decreased gradually with the increase of XBTYF concentration. Values represent the average of three replicates, ^*∗*^*P* < 0.05 vs. model. (b) Expression of apoptosis-related proteins Bax, Bcl2, caspase 3, and caspase 9 was assessed by western blot, and the expression of Bax, caspase 3, and caspase 9 was significantly decreased, while Bcl2 expression was increased compared to the model. Values represent the average of three replicates, ^*∗*^*P* < 0.05 vs. model.

To examine whether XBTYF exerts an effect on cell apoptosis, we performed TUNEL staining to measure cell apoptosis and western blot to detect the expression of Bax, Bcl2, caspase 3, and caspase 9. All data are normally distributed (*P* > 0.05), and we perform statistical analysis using one-way ANOVA. The results in Figure 4(a) revealed that degree of cell apoptosis in the model rats was higher than that in XBTYF-treated rats (*P* < 0.05). With the increase in XBTYF concentration, cell apoptosis decreased gradually. The levels of Bax, caspase 3, and caspase 9 in the model group were much higher compared to those in the control, sham-operated, high-, medium-, and low-dose XBTYF groups, whereas Bcl2 expression was decreased (Figure 4(b)).

### 3.5. XBTYF Promoted Angiogenesis via VEGF-Notch1/Dll4 Pathway

**Figure 5:**
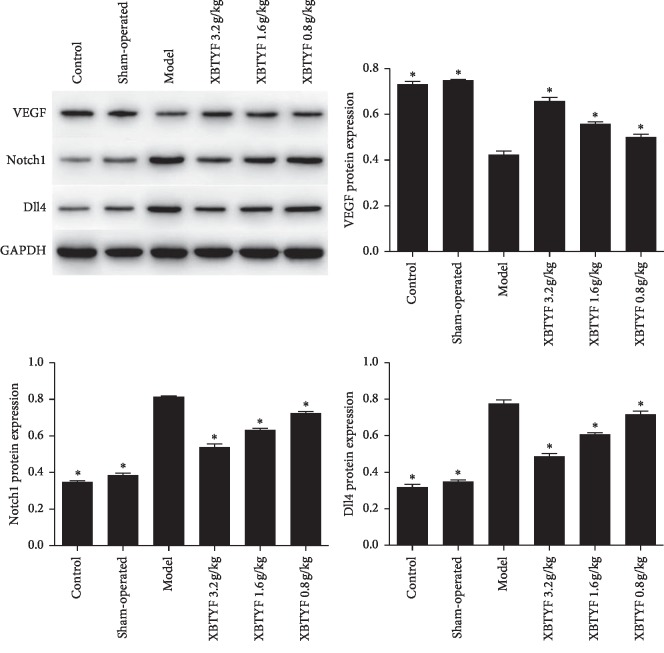
Protein expression of VEGF, Notch1, and Dll4 was measured by western blot. Compared with the model group, the expression of Notch1 and Dll4 was significantly decreased by XBTYF at all doses (3.2, 1.6, and 0.8 g/kg), whereas that of VEGF was increased. Values represent the average of three replicates, ^*∗*^*P* < 0.05 vs. model.

We measured the expression of VEGF-A, Notch1, and Dll4 to evaluate whether XBTYF promotes angiogenesis through the VEGF-Notch1/Dll4 pathway. The expression data of VEGF-A, Notch1, and Dll4 were normally distributed (*P* > 0.05). From Figure 5, we observed that compared with the model group, the expression of Notch1 and Dll4 was significantly decreased by XBTYF at all doses, whereas that of VEGF was increased. High-dose XBTYF (3.2 g/kg) had the greatest effect among all XBTYF groups.

## 4. Discussion

Myocardial ischemia refers to a decrease in blood perfusion in the heart, resulting in conditions such as hypoxia and abnormal energy metabolism [15, 16]. CHD is the main cause of myocardial ischemia. With the improvement of living standards, the prevalence of myocardial ischemia in China is increasing annually and it has become a frequently occurring disease in elderly people. In recent years, studies on the pathological mechanisms of myocardial ischemic injury have been highlighted in myocardial ischemia research [17–20]. Treatments based on traditional Chinese medicine have shown potential applications in myocardial ischemia treatment, and this has been reported in many studies. Ji [21] found that supplementing qi and activating blood circulation clearly improved the clinical symptoms of myocardial ischemia. Wu and Liu [22] observed that Danggui Buxue Tang protected rats against myocardial ischemia by reducing the expression of inflammatory factors. Chen et al. [23] indicated that Shuangshen Tongmai Granules reduced the expression of oxidative stress-related factors and the apoptotic rate of cardiomyocyte. XBTFY is a well-known traditional Chinese medical formulation that has been widely used in clinical settings [24]. Our previous experiments revealed that XBTYF protected the heart of rats with myocardial ischemia by promoting angiogenesis [12, 13], but the underlying mechanism of action involved therein remains unclear.

Wang et al. [25] found that crocetin protected cardiomyocyte by upregulating the apoptosis-inhibitory protein Bcl2 and downregulating the proapoptotic protein Bax. Leng et al. [26] indicated that the protective effect of Rb1 on the ischemic myocardium may be related to its inhibition of cell apoptosis. In this study, we used HE and TUNEL staining to examine the effect of XBTYF on myocardial cell apoptosis. The results showed that XBTYF reduced cell apoptosis and morphological abnormalities in cardiomyocyte. We further detected the expression levels of apoptosis-related proteins Bax, Bcl2, caspase 3, and caspase 9. The results support the previous conclusions, indicating that XBTYF effectively reduced cardiomyocyte apoptosis.

Mitochondria play an important role in myocardial ischemia. Adenosine triphosphate (ATP) is the main form of energy used by the heart, but because the energy reserve of the heart is very small, ATP must be synthesized efficiently and promptly [27]. When the structure and function of mitochondria become abnormal, cardiomyocyte apoptosis will be induced [28]. Dysfunction of energy metabolism in myocardial cells is an important pathological basis of myocardial ischemia [27], and maintaining the functional stability of mitochondria in myocardial cells and the metabolic balance of ATP can effectively alleviate myocardial injury [29, 30]. He and He [31] found that resveratrol reduced mitochondrial damage in myocardial cells, inhibited inflammatory reaction, and reduced myocardial cell apoptosis. Fan et al. [32] observed that astragalus polysaccharide reduced calcium overload in cardiomyocytes, decreased mitochondrial membrane potential, suppressed reactive oxygen species production, and played a role in protecting cardiomyocyte. In this study, we observed the effect of XBTYF on mitochondria and found that it reduced damage to mitochondrial structure and exerted protection on mitochondrial function. We also showed that the expression of Cyt-c in myocardial cells decreased significantly after XBTYF administration, further suggesting that XBTYF protected the structure and function of mitochondria from damage.

In the early stage of myocardial ischemia, necrotic cardiomyocytes release a series of angiogenic factors, promote the formation of neovascularization around blocked or narrowed vessels, form new bypass circulation, and compensatively alleviate the phenomenon of myocardial ischemia. However, the increase in endogenous angiogenesis-promoting substances is usually not enough to establish sufficient collateral circulation to compensate for the original blood supply, and myocardial ischemia cannot be effectively alleviated. Therefore, researchers have proposed that in vitro intervention can promote the secretion of vascular growth factors and the upregulation of related receptors, thereby establishing effective collateral circulation to meet the needs of heart blood supply. Li et al. [33] showed that both VEGF and angiotensin-1 promoted angiogenesis. Wu et al. [34] revealed that Yiqi Huoxue recipe downregulated the expression of hypoxia-inducible factor-1*α* and VEGF receptor-2 and promoted angiogenesis in rats with myocardial ischemia. Wang et al. [35] found that Xuefu Zhuyu Tang increased the expression of VEGF and promoted angiogenesis in ischemic myocardium. Zhang [36] observed that Tongxinluo promoted angiogenesis in cardiomyocytes and upregulated the expression of VEGF and basic fibroblast growth factor. Yang et al. [37] suggested that butylphthalide could promote angiogenesis by activating the VEGF/Notch1/Dll4 signaling pathway. In this experiment, changes in the expression levels of VEGF, Notch1, and Dll4 were detected by western blot. It was revealed that XBTYF reduced the protein expression levels of Notch1 and Dll4 and promoted the expression of VEGF-A, indicating that XBTYF promoted angiogenesis through the VEGF-Notch1/Dll4 pathway. Amani et al. [38] revealed that many inflammatory and metabolic signaling pathways are involved in cell fate following ischemia/reperfusion injury, such as hippo, ubiquitin-proteasome system (ERK5), Tsc1/Tsc2 complex, FoxO1, wnt/*β*-catenine, Adamts1, and Jak2/stat3 signaling pathway. Whether these signaling pathways can also be activated by XBTYF needs to be further investigated.

## 5. Conclusion

XBTYF attenuated mitochondrial damage and cell apoptosis and promoted angiogenesis of cardiomyocyte, and the mechanism may be related to the VEGF-Notch1/Dll4 pathway. Thus, we suggest that XBTYF could be a potentially effective agent for the treatment of myocardial ischemia.

## Data Availability

The data used to support the findings of this study are available from the corresponding author upon request.
